# Rapid effects of extrafine beclomethasone dipropionate/formoterol fixed combination inhaler on airway inflammation and bronchoconstriction in asthma: a randomised controlled trial

**DOI:** 10.1186/1471-2466-11-60

**Published:** 2011-12-21

**Authors:** Brian J O'Connor, Sara Collarini, Gianluigi Poli, Caterina Brindicci, Monica Spinola, Daniela Acerbi, Peter J Barnes, Brian Leaker

**Affiliations:** 1 Heart Lung Centre, Respiratory Clinical Trials Ltd, Queen Anne Street Medical Centre, 18-22 Queen Anne Street, London W1G 8HU, UK; 2 Chiesi Farmaceutici S.p.A., Via Palermo 26/A, 43122 Parma, Italy; 3 National Heart & Lung Institute, Imperial College, Exhibition Rd, London SW7 2AZ, UK

## Abstract

**Background:**

The dose-dependent anti-inflammatory effects of a recent fixed combination of extrafine beclomethasone dipropionate/formoterol (BDP/F) were investigated using non-invasive markers of inflammation, exhaled nitric oxide (NO) and adenosine monophosphate (AMP) provocative challenge. The aim was to assess the onset of the anti-inflammatory action of low and high doses and evaluate the suitability of non-invasive assessments to demonstrate dose response.

**Methods:**

Steroid naïve adult out-patients with mild asthma, sensitive to AMP with baseline exhaled NO > 25 parts per billion entered a double-blind, placebo-controlled, 3-way, cross-over study. Patients were randomised to low dose (1 actuation) or high dose (4 actuations) extrafine BDP/F 100/6 μg, or placebo administered twice daily on Days 1 and 2 and once in the morning on Day 3 of each period. Exhaled NO was measured pre-dose on Day 1, then 2 and 4 hours post-administration on Day 3. The AMP challenge was performed 4 hours post-administration on Day 3 and forced expiratory volume in 1 second (FEV_1_, L) was measured from 0 to 4 hours post-dose on Day 1. Endpoints were NO at 2 and 4 hours, AMP challenge at 4 hours after the fifth dose on Day 3 and FEV_1 _area under the curve from 0 to 4 h post-dose on Day 1. Analysis of covariance was performed for NO and FEV_1 _and analysis of variance for AMP challenge.

**Results:**

Eighteen patients were randomised and completed the study. Exhaled NO was significantly lower for both doses of extrafine BDP/F versus placebo at 2 and 4 hours (high dose LS mean difference: -22.5 ppb, p < 0.0001 and -20.5 ppb, p < 0.0001; low dose: -14.1 ppb, p = 0.0006 and -12.1 ppb, p = 0.0043) with a significant dose response (p = 0.0342 and p = 0.0423). Likewise, AMP challenge revealed statistically significant differences between both doses of extrafine BDP/F and placebo (high dose LS mean difference: 4.8 mg/mL, p < 0.0001; low dose: 3.7 mg/mL, p < 0.0001), and a significant dose response (p = 0.0185). FEV_1 _was significantly improved versus placebo for both doses (high dose LS mean difference: 0.2 L, p = 0.0001; low dose: 0.2 L p = 0.0001), but without a significant dose response.

**Conclusions:**

The fixed combination inhaler of extrafine BDP/F has early dose-dependent anti-inflammatory effects with a rapid onset of bronchodilatation in mild asthmatic patients.

**Trial Registration:**

ClinicalTrials.gov: NCT01343745

## Background

Asthma is a chronic inflammatory disease of the whole bronchial tree. Persistent inflammation of the airways and increased bronchial reactivity has been recognised even in mild asthma [1]. Current guidelines [2] suggest a step-wise approach starting with inhaled corticosteroids (ICS), the mainstay of asthma therapy and the most effective anti-inflammatory treatment available for persistent asthma. Corticosteroids control airway inflammation and decrease bronchial hyperreactivity (BHR), thus reducing asthma symptoms and improving lung function [3]. Maximal clinical benefits from corticosteroids are expected within weeks [4] but recent evidence suggests that ICS can exert acute effects (within hours) on airways inflammation and BHR [5-7].

There are, however, systemic side-effects at high doses of ICS [8] and, in patients uncontrolled on a medium doses of ICS, addition of an inhaled long-acting β_2_-agonist (LABA) is the preferred therapy. These two classes of drugs address complementary aspects of the pathophysiology of asthma in terms of anti-inflammatory and bronchodilating effects that neither class is able to achieve alone [9]. On addition of a LABA, improvements are seen in symptom scores and lung function with a decrease in the number of exacerbations. The use of a LABA with an ICS achieves clinical control in more patients at a lower dose of the ICS than if the ICS were used alone [10,11].

A fixed combination of beclomethasone dipropionate (BDP) and formoterol (F) (Foster^®^, Chiesi Farmaceutici), delivered via a hydrofluoroalkane (HFA), pressurised metered dose inhaler (pMDI), is characterised by an extrafine particle formulation. This extrafine particle formulation ensures uniform delivery of the two active drugs to large and small airways, therefore treatment of inflammation and bronchoconstriction is expected throughout the whole bronchial tree [12].

The accurate assessment of the effect of treatment on airways inflammation in asthma is important for successful clinical management of the disease. There is a growing interest in non-invasive markers of airways inflammation, e.g. exhaled nitric oxide (NO) which could be used in addition to traditional methods, such as lung function tests and symptom scores, to monitor early deterioration of lung function. Raised levels of fractional exhaled nitric oxide (FE_NO_) are associated with inflammation in asthma, are responsive to suppression by corticosteroids and there is also evidence of its association with asthma severity. This makes exhaled NO a sensitive and practical surrogate marker to monitor ICS treatment effect [13-15].

Bronchial hyperreactivity is present in virtually all patients with asthma and is an indirect marker of airway inflammation. A bronchoconstrictor stimulus to measure BHR is adenosine monophosphate (AMP) which acts via release of histamine and other mediators from mast cells. The concentration of AMP causing the forced expiratory volume in 1 second (FEV_1_) to decrease by 20% (AMP PC_20_) may be used as a non-invasive marker of airways inflammation and bronchoprotective effects on AMP have been detected with both ICS and LABA [16,17].

The FEV_1 _is a lung function parameter which is commonly used for monitoring the level of airways obstruction and an improvement in FEV_1 _after treatment with β-agonists is well documented [18].

There is limited literature available on acute effects of ICS treatment on surrogate markers of inflammation. The models used so far have sometimes failed to show dose-response effects and have never been tested in ICS/LABA fixed combinations, whose use in asthma management is continuously increasing. The objective of this exploratory study was to assess the onset of the anti-inflammatory action of a fixed combination of extrafine BDP/F at increasing doses (low dose: 100/6 μg, 1 actuation; high dose: 100/6 μg, 4 actuations) and also to evaluate the suitability of non-invasive assessments (FE_NO_, AMP PC_20 _and FEV_1_) to demonstrate a dose response. AMP challenge has proved to be a useful marker of corticosteroid anti-inflammatory activity but it is known to be influenced also by the LABA component. For this reason, we included also measurement of FE_NO _levels, which are specifically affected by ICS only, in order to discriminate the contribution of the two components of the fixed combination.

## Methods

### Study subjects

Patients were aged 18 to 50 years with clinical evidence of asthma (associated with either demonstration of ≥ 12% reversibility and 200 mL improvement of FEV_1 _using a standard dose of salbutamol within 30 minutes, or historical BHR to methacholine, within 12 months of the screening visit). They had to be steroid naïve (i.e., patients should have never taken steroid medications before) with FEV_1 _> 70% of predicted value and at least 2.0 L at screening. Intake of any anti-asthmatic drug had to be stopped before the study entry, with the exception of inhaled salbutamol as rescue medication, which was allowed during the study but for the 8 h prior to lung function measurements. Patient's asthma had to be stable, without experiencing any respiratory tract infection or any exacerbation requiring treatment with oral steroid in the 4 weeks prior to the study entry. Patients were non-smokers or ex-smokers (< 5 pack-years). Patients had a body mass index (BMI) between 18 and 35 kg/m^2^. Patients had to be sensitive to AMP (PC_20 _after the AMP challenge test at screening ≤ 20 mg/mL) and have baseline FE_NO _levels > 25 parts per billion (ppb). Patients with history of cystic fibrosis or bronchiectasis or alpha-1 antitrypsin deficiency or any other clinically significant lung disease, including COPD, were not included in the study. The study was carried out in accordance with the Declaration of Helsinki, the ICH Harmonised Tripartite Guideline for Good Clinical Practice (GCP), and with applicable regulatory requirements. The study protocol was approved by an independent ethics committee (the St Thomas' Research Ethics Committee London, United Kingdom).

### Study design

**Figure 1:**
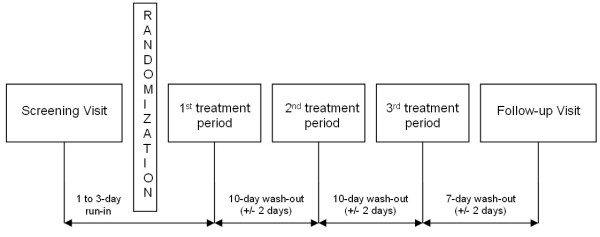
**Study design**.

This was a single centre study of randomised, double-blind, double-dummy, placebo controlled, 3-way multiple dose, cross-over design (Figure 1). Treatments were randomly assigned (3 treatments, 6 sequences) and the randomisation list was not accessible to patients, investigators, monitors or employees of the clinical site and the sponsor's clinical team, unless in case of emergency. Patients were recruited from the clinical site database and by advertising. After screening, eligible patients entered the study, which comprised three 3-day treatment periods each separated by a 10-day wash-out period. The period of wash-out was chosen according to available literature [5,19] and it is in line with the pharmacokinetic properties of BDP, showing an elimination half-life of about 3 hours for the active metabolite 17-BMP [20]. The 3 treatments administered were:

• low dose extrafine BDP/F (Foster^®^; Chiesi Farmaceutici): 100/6 μg, 1 actuation

• high dose extrafine BDP/F: 100/6 μg, 4 actuations

• placebo pMDI.

The order in which each patient received the treatments was randomised. Study treatments were administered twice daily on Days 1 and 2, and once, in the morning, on Day 3 (a total of 5 doses). Before taking the study medication, patients were trained to the use of the pMDI inhaler with Vitalograph^® ^Aerosol inhalation monitor. No spacer was used for drug administration during the study.

The efficacy endpoints were:

• FE_NO _levels at 2 and 4 hours after the fifth dose on Day 3 of each study period

• AMP PC_20 _at 4 hours after the fifth dose on Day 3 of each study period

• normalised by time-area under the curve between 0 and 4 hours (AUC_0-4h_) after the morning administration FEV_1 _measured on Day 1 of each study period.

### Measurement of FE_NO_

Standardised FE_NO _measurements were performed using the NIOX^® ^(Aerocrine, Solna, Sweden) analyser. NO was measured at 50 mL/s expiratory rate, according to standard procedures [21,22]. The average of two acceptable values was considered for the statistical analysis. FE_NO _measurements were performed at screening and during each treatment period on Day 1 pre-dose, and 2 and 4 hours post-administration on Day 3. In case of concomitant assessments, these measurements were conducted immediately before the AMP challenge test.

### AMP challenge test

The AMP challenge test was performed at screening and on Day 3 of each treatment period 4 hours post-administration, according to a standardized challenge protocol as previously described [23]. The highest of 3 FEV_1 _recordings taken before administration of the diluent was used as the pre-saline baseline. The challenge was not carried out if FEV_1 _was < 60% predicted or the patient manifested significant asthma symptoms of wheeze, chest tightness or cough. Patients inhaled 0.9% saline, nebulised from a breath-activated dosimeter of known output. The higher of two measurements taken after inhalation of saline was used as the post-diluent FEV_1 _to calculate the PC_20 _value. Patients then inhaled doubling increments of AMP until a ≥ 20% fall in FEV_1 _from the post-saline value was achieved or the maximum concentration had been given. If the highest FEV_1 _between the two duplicates was < 20% below the post-saline FEV_1 _reference, patients progressed to the next highest concentration of AMP. Doubling concentrations of AMP ranging from 0.16 to 640 mg/mL were used.

### Measurement of FEV_1_

At screening, FEV_1 _was measured before and after administration of salbutamol for the reversibility test. During each treatment period, FEV_1 _area under the curve (AUC) was calculated from 0 to 4 h post-dose on Day 1 by measuring FEV_1 _pre-dose and 0.5, 1, 2 and 4 hours post-administration according to American Thoracic Society/European Respiratory Society standards [24]. For Caucasians of non-European descent and non-Caucasians, predicted values for FEV_1 _and forced vital capacity (FVC) were to be adjusted for race as per the European Coal and Steel Community (ECSC) guidelines [25]. Values were corrected for BTPS conditions (saturated with water vapour at body temperature [37°C] and at the ambient barometric pressure). The rescue medication (salbutamol) was to be withheld for at least 8 hours prior to administration of each dose of study medication.

### Safety assessments

Evaluation of the safety profile included collection and monitoring of any adverse events (AEs) throughout the study. Vital signs (heart rate and blood pressure) were measured at screening and in each treatment period before and after drug administration and routine clinical laboratory assessments were made at screening and at the end of the study.

### Statistical analysis

Due to the exploratory nature of this study, no formal sample size calculation was made. Efficacy data were analysed for the intention-to-treat (ITT) population as well as the per-protocol (PP) population. The ITT population included all randomised patients who received at least one inhalation of study drug and had at least one post-baseline efficacy evaluation. The PP population included all patients from the ITT population without any major protocol violation (i.e. wrong inclusion, poor compliance, intake of forbidden medication, etc). Efficacy data from the ITT and PP populations were comparable. Therefore, only the results from the ITT population are reported in the current paper. Safety was assessed in all randomised patients who received at least one inhalation of study medication.

Comparison between treatments for FE_NO _and FEV_1 _AUC(0-4h) was carried out using an analysis of covariance (ANCOVA) for a cross-over design with patient (sequence and patient within sequence), period and treatment as factors of the model and pre-dose values on each treatment period as covariate.

The following rule was applied for PC_20 _calculation when patients did not reach a 20% fall in FEV_1_: an AMP concentration of 640 mg/mL was used for patients with 10% or greater FEV_1 _fall; an AMP concentration of 1280 mg/mL (twice the maximum concentration level of AMP administered) was used for patients with an FEV_1 _fall less than 10%. A logarithmic transformation using base 2 (Log2) was applied before analysis for PC_20 _(doubling doses scale). Comparison between treatments for PC_20 _was carried out using an analysis of variance (ANOVA) for a cross-over design with patient (sequence and patient within sequence), period and treatment as factors of the model.

For each comparison, the least square (LS) mean, pairwise treatment effect, the 95% confidence interval (CI) and the probabilities (p-values) were shown.

## Results

Of 36 patients screened, 18 (10 males and 8 females) were randomised, received study medication and all completed the study. All patients gave their written informed consent before any study related procedure. Randomised patients had a median age of 28.5 years (range: 19-46 years). The other demographic characteristics and baseline data for the study population are provided in Table 1. Patients had a history of mild asthma for a median period of 21 years (range: 4-46 years) and were steroid naïve. Seventeen out of 18 randomised patients were taking salbutamol as rescue medication for asthma before the study entry. At screening, median FEV_1 _was 3.4 L (98.5% of predicted) and median FVC was 4.4 L (111.0% of predicted). The efficacy results for the endpoints assessed in this study (FE_NO_, AMP PC_20_, and FEV_1_) are presented in Table 2 and the statistical comparisons are shown in Table 3.

**Table 1:** Patients' characteristics and baseline data.

Characteristic	Mean ± SD	Median (Range)
Age (years)	30.6 ± 9.2	28.5 (19-46)

FEV_1 _(L)	3.4 ± 0.7	3.4 (2.2-4.5)

% FEV_1 _predicted	94.9 ± 10.5	98.5 (71-111)

FVC (L)	4.4 ± 1.1	4.4 (2.8-6.2)

% FVC predicted	105.3 ± 13.2	111.0 (65-118)

FE_NO _(ppb)	72.4 ± 27.1	82 (27-120)

AMP PC_20 _(mg/mL)	6.5 ± 6.0	4.5 (0.4-19.3)

SD = standard deviation; FEV_1 _= forced expiratory volume in one second; FVC = forced vital capacity; FE_NO _= Fractional exhaled nitric oxide; AMP PC_20 _= AMP challenge provocative concentration causing a 20% fall in FEV_1_

**Table 2:** Efficacy endpoints: FE_NO_, PC_20 _AMP, and FEV_1_.

FE_NO _(ppb) - Day 3, 4 hours	LS Mean	95% CI
High dose	50.5	43.8, 57.3

Low dose	58.9	52.2, 65.7

Placebo	71.1	64.4, 77.8

**FE_NO _(ppb) - Day 3, 2 hours**	**LS Mean**	**95% CI**

High dose	51.8	44.5, 59.2

Low dose	60.2	53.0, 67.5

Placebo	74.4	67.2, 81.5

**AMP PC_20 _(mg/mL) - Day 3, 4 hours**	**Log2 LS Mean**	**Log2 95% CI**

High dose	7.1	5.9, 8.2

Low dose	6.0	4.9, 7.1

Placebo	2.3	1.2, 3.4

**FEV_1 _(L) - AUC _0-4 h _Day 1**	**LS Mean**	**95% CI**

High dose	3.6	3.5, 3.6

Low dose	3.6	3.5, 3.6

Placebo	3.4	3.3, 3.4

LS Mean = least squares mean; CI = confidence interval; FE_NO _= Fractional exhaled nitric oxide; AMP PC_20 _= AMP challenge provocative concentration causing a 20% fall in FEV_1_; FEV_1 _= forced expiratory volume in one second.

**Table 3:** Statistical comparisons of efficacy endpoints.

FE_NO _(ppb) - Day 3, 4 hours	LS Mean	95% CI	P-value
High dose - placebo	-20.5	-28.5, -12.6	< 0.0001

Low dose - placebo	-12.1	-20.1, -4.1	0.0043

High dose - low dose	-8.4	-16.6, -0.3	0.0423

**FE_NO _(ppb) - Day 3, 2 hours**	**LS Mean**	**95% CI**	**P-value**

High dose - placebo	-22.5	-30.1, -15.0	< 0.0001

Low dose - placebo	-14.1	-21.7, -6.6	0.0006

High dose - low dose	-8.4	-16.2, -0.7	0.0342

**AMP PC_20 _(mg/mL) - Day 3, 4 hours**	**Log2 LS Mean**	**Log2 95% CI**	**P-value**

High dose - placebo	4.8	3.9, 5.6	< 0.0001

Low dose - placebo	3.7	2.8, 4.6	< 0.0001

High dose - low dose	1.0	0.2, 1.9	0.0185

**FEV_1 _(L) - AUC _0-4 h _Day 1**	**LS Mean**	**95% CI**	**P-value**

High dose - placebo	0.2	0.1, 0.3	0.0001

Low dose - placebo	0.2	0.1, 0.3	0.0001

High dose - low dose	0.0	-0.1, 0.1	0.9832

LS Mean = least squares mean; CI = confidence interval; FE_NO _= Fractional exhaled nitric oxide; AMP PC_20 _= AMP challenge provocative concentration causing a 20% fall in FEV_1_; FEV_1 _= forced expiratory volume in one second.

### FE_NO_

**Figure 2:**
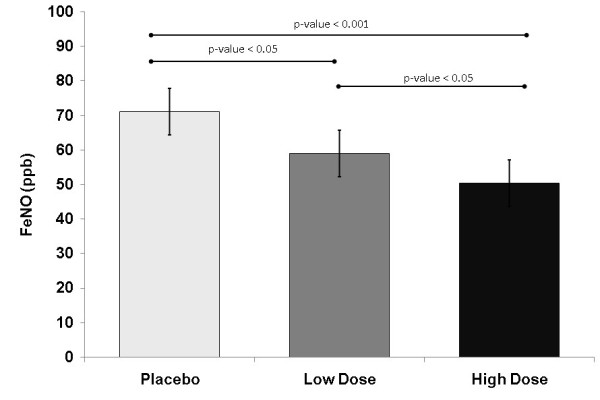
**FE_NO _levels at 4 hours post-administration after 3 days of treatment with extrafine BDP/F low dose (100/6 μg, 1 actuation), high dose (100/6 μg, 4 actuations), or placebo**. Columns and bars represent LS mean and 95% CI.

The FE_NO _at 2 hours post-treatment on Day 3 was significantly lower after both doses of extrafine BDP/F compared with placebo (high dose comparison LS mean difference: -22.5 ppb, p < 0.0001; low dose comparison: -14.1 ppb, p = 0.0006, respectively) (Table 3). The results were similar at 4 hours post-treatment on Day 3 with FE_NO _significantly lower after both doses of extrafine BDP/F compared with placebo (high dose comparison: -20.5 ppb, p < 0.0001; low dose comparison: -12.1 ppb, p = 0.0043, respectively) (Table 3). At both time points a significant dose response was confirmed (high vs low dose comparison: -8.4 ppb; p = 0.0342 and p = 0.0423 at 2 and 4 hours, respectively). Figure 2 shows the LS mean FE_NO _at 4 hours on Day 3 for each treatment group.

### PC_20 _AMP

**Figure 3:**
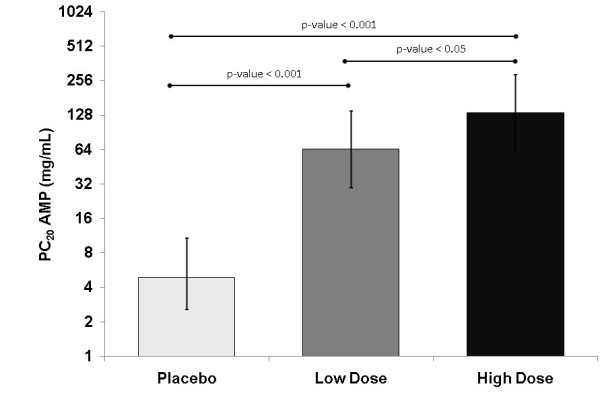
**AMP PC_20 _at 4 hours post-administration after 3 days of treatment with extrafine BDP/F low dose (100/6 μg, 1 actuation), high dose (100/6 μg, 4 actuations), or placebo**. Columns and bars represent LS mean and 95% CI.

The AMP PC_20 _on Day 3 was significantly higher after both doses of BDP/F compared with placebo (high dose comparison LS mean difference: 4.8, p < 0.0001; low dose comparison: 3.7, p < 0.0001) (Table 3). A significant dose response was confirmed between high and low dose (p = 0.0185) with a difference of 1.0 in AMP PC_20 _on doubling the dose concentration. Figure 3 shows the LS mean AMP PC_20 _for each treatment group. Few patients showed FEV_1 _values < 60% predicted after the last administered provocative concentration of AMP, but they did not manifest any asthma symptom.

### FEV_1_

**Figure 4:**
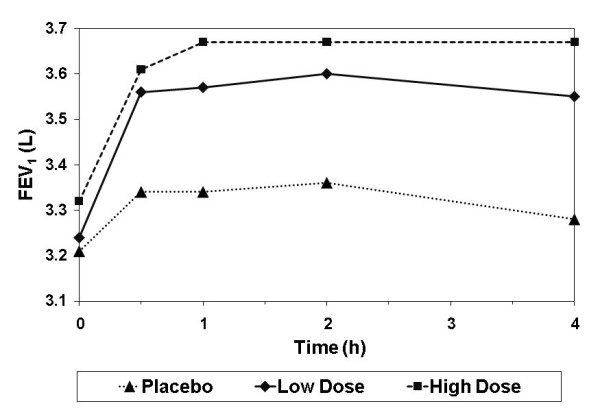
**Mean FEV_1 _on the first day of treatment with extrafine BDP/F low dose (100/6 μg, 1 actuation), high dose (100/6 μg, 4 actuations), or placebo**.

The AUC_0-4h _using FEV_1 _(L) on Day 1 was significantly higher after both doses of BDP/F compared with placebo (high dose comparison LS mean difference: 0.2, p = 0.0001; low dose comparison: 0.2, p = 0.0001) (Table 3). No significant dose response was confirmed. Figure 4 shows the LS mean FEV_1 _over time on Day 1 for each treatment group.

### Safety

The rate of adverse events (AEs) reporting was similar for each treatment. Adverse events were reported by 7 patients (39%) during placebo treatment, 7 (39%) during low dose extrafine BDP/F treatment, and 8 (44%) during high dose extrafine BDP/F treatment. Most AEs were recorded as not related to study medication. There were no deaths, serious AEs or withdrawals due to AEs. There were also no clinically significant laboratory results and no clinically significant changes in vital signs.

## Discussion

Several studies have reported that ICS can exert a rapid anti-inflammatory activity within few days from the beginning of treatment [5-7]. Results have shown rapid improvement of surrogate markers of inflammation, such as FE_NO _and AMP PC_20_, but these effects were not always dose-dependent [26,7,6]. To our knowledge, this is the first study assessing the acute anti-inflammatory activity of an ICS/LABA fixed combination on two different surrogate endpoints, i.e. FE_NO _and PC_20 _to AMP challenge, and at two different dose levels. In addition to confirming previous results on acute effects of ICS, these findings provide evidence of a useful model to be used for comparative studies. Reference guidelines require equivalence between inhalation drugs to be studied by comparison of at least two different doses and after 4 weeks of treatment [27]. With this study, we have shown that significant effects can be already detected after 3 days of treatment with an ICS/LABA fixed combination and that the model is dose sensitive.

Aalbers et al. [19] conducted a placebo-controlled study comparing one single dose of budesonide/formoterol fixed combination against formoterol alone and against salbutamol alone. The study measured only changes in airways responsiveness to AMP challenge, which is a useful marker of corticosteroid anti-inflammatory activity but also of the effects of the LABA component [16,17]. Greater effects were seen with the fixed combination at 3 and 7 hours after inhalation compared with either β_2_-agonists alone, supporting an additive effect of ICS in protecting against AMP-induced bronchoconstriction. In contrast to the study of Aalbers et al. [19], our study also included measurement of a surrogate marker specifically affected by ICS only, i.e. FE_NO _levels, in order to distinguish the contribution of the ICS from that of the LABA component in determining the acute response to BDP/F.

The efficacy parameters all demonstrated statistically significant differences between both doses of BDP/F (BDP/F 100/6 μg, 1 actuation vs 4 actuations) and placebo. In addition, FE_NO _at 2 and 4 hours on Day 3 and PC_20 _after AMP tests demonstrated statistically significant differences between the 2 dose levels of study drug.

FE_NO _reflects eosinophilic airways inflammation and various studies have provided sufficient data to justify the use of FE_NO _to identify and monitor steroid response as well as steroid requirements in the diagnosis and management of asthma. Kharitonov and Barnes [13] measured FE_NO _levels in 28 patients with mild asthma who were administered 400 μg budesonide, 100 μg budesonide or placebo once daily for 3 weeks followed by 1 week without treatment in a parallel group study. A dose-dependent speed of onset and cessation of action of budesonide was seen on FE_NO _and asthma symptoms. However, no changes compared with baseline were seen early at 3 and 6 hours after single dose; changes were noted only from Day 3 and these were dose-dependent. Similarly, our experimental model, utilising several outcome measures and early post-treatment observations, enabled us to see significant acute dose-response reduction of FE_NO _after 3 days of treatment with an extrafine ICS/LABA fixed combination.

Erin et al. [7] investigated the rapid effect of inhaled ciclesonide on FE_NO _levels in 21 mild asthmatics administered ciclesonide 320 μg once daily, ciclesonide 640 μg twice daily or placebo for 7 days in a double blind cross-over study. Exhaled NO was assessed on Days 1, 3 and 7 after inhalation of study drug. Both ciclesonide 320 μg once daily and 640 μg twice daily produced significantly reduced exhaled NO levels from Day 3 compared with placebo but a dose-dependent response was not seen. Ciclesonide is known to be clinically effective at doses lower than those used in the study reported, so the doses administered in this study may have been too high to detect any dose-dependent response. The study by Erin et al. [7] also investigated the effects of ciclesonide on AMP PC_20 _challenge test. Both doses of ciclesonide significantly reduced airways responsiveness compared with placebo from Day 1, and a dose-dependent response was not seen. Another study [28] reported dose-related improvement of PC_20 _to AMP challenge in 29 patients with mild to moderate allergic asthma treated with ciclesonide 100 μg, 400 μg and 1600 μg daily. However, in this study patients underwent a longer treatment of 14 days and no measurement was taken to assess short-term changes in AMP PC_20_. The study by Taylor et al. [23] also observed a significant reduction in the percentage of eosinophils in induced sputum for 400 μg and 1600 μg ciclesonide daily but this was not dose-dependent.

Ketchell et al [6] investigated the rapid effect of inhaled fluticasone propionate therapy (100, 250, or 1000 μg) on airway responsiveness in 3 consecutive cross-over studies in steroid naïve subjects with mild asthma. The results showed that fluticasone significantly reduced airway responsiveness to AMP in comparison with placebo after 3 and 7 inhalations and a trend towards a greater effect with a higher dose was observed, although the difference did not achieve statistical significance. In these studies, the AMP challenge test was performed 2 hours after inhalation differently from other studies where AMP PC_20 _was measured at 4 hours. The time interval between drug administration and AMP challenges may have been too short to detect any significant dose-related change.

There is limited literature available on the acute effects of ICS treatment on surrogate markers of inflammation in asthma. These studies reported significant improvement of FE_NO _levels and BHR, but in most cases they did not demonstrate the dose-dependent nature of the response to steroid treatment.

Our study design was a 3-way cross-over study with 3 days of treatment and 10 days washout. This model allowed discrimination of rapid and dose-related differences on two different markers of inflammation after 5 inhalations of study drug. By combining the use of AMP PC_20_, which is an index of the effects of ICS and LABA, and FE_NO_, which is affected by ICS only, we were able to show that the ICS component of extrafine BDP/F fixed combination can exert a rapid anti-inflammatory activity. The dose response observed for FE_NO _is driven by the ICS component of the fixed combination, as it is well known that formoterol monotherapy has no effects on exhaled NO. With regard to the dose response observed for AMP PC_20_, it is recognized that formoterol exhibited antagonism against AMP challenge to a lesser degree than ICS [29]. However, it is our opinion that dose response observed for AMP PC_20 _is likely due to both components of the fixed combination. No difference was found in FEV_1 _values between low and high dose BDP/F. This was to be expected because the study involved steroid naïve patients in order to avoid confounding factors that could have masked the effect of the ICS in the study drug. The treatment was well tolerated in this study population.

The limitations of our study are related to the small sample size and to the possible overlapping effects of the ICS and LABA components. Due to the exploratory nature of the study it was not possible to make a formal sample size calculation. However, despite the reduced number of enrolled patients, we were able to identify significant improvement of airway inflammation and to detect a dose-response effect. As far as the AMP challenge test is concerned, it is known that this parameter is not specific for ICS only but can be affected also by the LABA included in the fixed combination. To overcome this limitation, we measured also the levels FE_NO_, which is specifically affected by ICS only.

## Conclusions

In conclusion, we demonstrated that extrafine BDP/F (100/6 μg per actuation) has rapid anti-inflammatory effects and produces a prompt early bronchodilator effect in patients with mild asthma. Also, that surrogate markers of inflammation such as FE_NO _and AMP PC_20 _are useful to demonstrate early dose-dependent effects of treatment.

## List of abbreviations

AMP: adenosine monophosphate; AMP PC_20_: provocative concentration of AMP causing a 20% fall in FEV_1_; BDP/F beclomethasone dipropionate/formoterol; BHR: bronchial hyperreactivity; BTPS: body temperature and pressure saturated; FE_NO_: fractional exhaled nitric oxide; FEV_1_: forced expiratory volume in 1 second; FVC: forced vital capacity; HFA: hydrofluoralkane; ICS: inhaled corticosteroid; LABA: long-acting β_2_-agonist; NO: nitric oxide; pMDI: pressurised metered dose inhaler.

## Competing interests

PJ Barnes has served on Scientific Advisory Boards of AstraZeneca, Boehringer-Ingelheim, Chiesi Farmaceutici S.p.A., GlaxoSmithKline, Novartis, Pfizer, Teva and UCB and has received research funding from AstraZeneca, Boehringer-Ingelheim, Chiesi Farmaceutici S.p.A., Daiichi-Sankyo, GlaxoSmithKline, Mistubishi-Tanabe, Novartis and Pfizer.

S Collarini, G Poli, C Brindicci, M Spinola and D Acerbi are full time employees of Chiesi Farmaceutici S.p.A.

B Leaker and BJ O'Connor have no competing interests to declare.

## Authors' contributions

BJO and BL participated in the design of the study, collection and interpretation of data. SC helped in the coordination of the study, analysis and interpretation of data and critically revised the manuscript. GP and DA participated in the design of the study, analysis and interpretation of data and critically revised the manuscript. CB participated in the design of the study and interpretation of data. MS contributed to interpretation of data and preparation of the manuscript. PJB contributed to analysis and interpretation of data and critically revised the manuscript. All authors read and approved the final manuscript.

## Pre-publication history

The pre-publication history for this paper can be accessed here:

http://www.biomedcentral.com/1471-2466/11/60/prepub
